# KNIME workflow for retrieving causal drug and protein interactions, building networks, and performing topological enrichment analysis demonstrated by a DILI case study

**DOI:** 10.1186/s13321-022-00615-6

**Published:** 2022-06-13

**Authors:** Barbara Füzi, Rahuman S. Malik-Sheriff, Emma J. Manners, Henning Hermjakob, Gerhard F. Ecker

**Affiliations:** 1 Department of Pharmaceutical Sciences, University of Vienna, Vienna, Austria; 2 European Molecular Biology Laboratory, European Bioinformatics Institute, Hinxton, Cambridge, UK

**Keywords:** Data science, Network, Causality, DILI, Targets, Enrichment analysis

## Abstract

As an alternative to one drug-one target approaches, systems biology methods can provide a deeper insight into the holistic effects of drugs. Network-based approaches are tools of systems biology, that can represent valuable methods for visualizing and analysing drug-protein and protein–protein interactions. In this study, a KNIME workflow is presented which connects drugs to causal target proteins and target proteins to their causal protein interactors. With the collected data, networks can be constructed for visualizing and interpreting the connections. The last part of the workflow provides a topological enrichment test for identifying relevant pathways and processes connected to the submitted data. The workflow is based on openly available databases and their web services. As a case study, compounds of DILIRank were analysed. DILIRank is the benchmark dataset for Drug-Induced Liver Injury by the FDA, where compounds are categorized by their likeliness of causing DILI. The study includes the drugs that are most likely to cause DILI (“mostDILI”) and the ones that are not likely to cause DILI (“noDILI”). After selecting the compounds of interest, down- and upregulated proteins connected to the mostDILI group were identified; furthermore, a liver-specific subset of those was created. The downregulated sub-list had considerably more entries, therefore, network and causal interactome were constructed and topological pathway enrichment analysis was performed with this list. The workflow identified proteins such as Prostaglandin G7H synthase 1 and UDP-glucuronosyltransferase 1A9 as key participants in the potential toxic events disclosing the possible mode of action. The topological network analysis resulted in pathways such as recycling of bile acids and salts and glucuronidation, indicating their involvement in DILI. The KNIME pipeline was built to support target and network-based approaches to analyse any sets of drug data and identify their target proteins, mode of actions and processes they are involved in. The fragments of the pipeline can be used separately or can be combined as required.

**Supplementary Information:**

The online version contains supplementary material available at 10.1186/s13321-022-00615-6.

## Introduction

The one-drug one-target paradigm has shifted in recent years due to increasing evidence of drugs usually interacting with more than one protein [1]. Approved and highly successful drugs, such as metformin or imatinib, address multiple targets simultaneously [2]. Systems biology studies could reveal novel desired and undesired target profiles for drugs [3]. Systems toxicology as a sub-field of systems biology aims to shed light on the mechanism of toxic drugs and the perturbed pathways to gather more information on the unwanted events [4]. Most proteins also do not act alone, which increases the need for systems biology approaches [5]. As appropriate representation and understanding of drug-protein and protein–protein interactions can be challenging, network-based methods can provide reliable tools for understanding interaction data. In these approaches, the interactions are represented as graphs where the nodes symbolize the interacting participants and edges represent the interactions. These methods are used to understand drug targets` properties, identify disease-target connections [6] and provide insights into drug toxicity by identifying the main contributors of unwanted events [7].

A more in-depth understanding of compound-protein and protein–protein interactions can be achieved by integrating information on the exact type of interaction between compound-protein and protein–protein. Techniques utilizing this information can help decode the mode of action of the drug, and the mechanism of a disease or an unwanted event. These methods offer a holistic approach considering the system as a whole and can provide valuable information on promising target combinations or preferable and problematic pathways. In a recently published study, causal network models were used for identifying promising candidates for drug repurposing in relation to SARS-CoV-2 [8].

One method in the toolkit of network-based approaches is topological enrichment analysis (TEA), which leverages information on the topology of the analysed network and pathways [9]. In this method, pathways are represented as graphs; nodes are the corresponding pathway components (e.g. proteins), and the edges provide information about the interaction among the nodes (e.g. up-regulation). TEA uses topological information to calculate pathway enrichment. TEA based on interaction networks can be used as a base for identifying pathways connected to a particular group of drugs, toxicity, or disease [10].

In this study, a KNIME workflow was developed that provides the opportunity to (i) collect causal drug and protein data, (ii) filter for tissue-specific proteins, (iii) build networks, (iv) gather causal protein–protein data and (v) perform topological enrichment analysis, using openly available data and web services. KNIME is an open-source workflow management system with a graphical interface allowing users to build complex data science pipelines [11].

As a case study, compounds linked to Drug-Induced Liver Injury (DILI) and no DILI compounds from the FDA were analysed. Tissue-specific target profiles were generated for both groups, which allowed significant proteins for the DILI group to be identified. A network of these proteins was created, and causal protein–protein connections were obtained. Finally, TEA of the significant proteins was conducted. This study demonstrates the usability of the workflow by identifying important proteins and processes in connection to DILI.

## Methods

### Case study

As a case study, the analysis of DILIRank data was chosen. DILIRank is the updated benchmark dataset of Drug-Induced Liver Injury (DILI) compounds of the FDA [12]. The drugs are grouped into four categories according to their potential to cause DILI. Our analysis was carried out with 180 drugs that are most likely to cause DILI (category “mostDILI”) and with 272 drugs which are not linked to DILI (“noDILI”). In order to demonstrate the general applicability of the workflow, we also performed two short case studies for cardiotoxicity and for nephrotoxicity. In the cardiotoxicity case study, approved small molecules from ChEMBL with ATC Classification Level 2 “Cardiac therapy” were downloaded, toxic compounds were filtered out. As the toxic group, withdrawn cardiotoxic compounds were collected, also from ChEMBL. The final dataset consists of 30 non-toxic cardiac therapy, and 26 cardiotoxic drugs. For the nephrotoxicity case study, a dataset was created by combining withdrawn nephrotoxic compounds from ChEMBL with a recently published dataset of nephrotoxic compounds based-on the SIDER database [13]. The combined dataset contains 19 compounds.

**Fig. 1:**
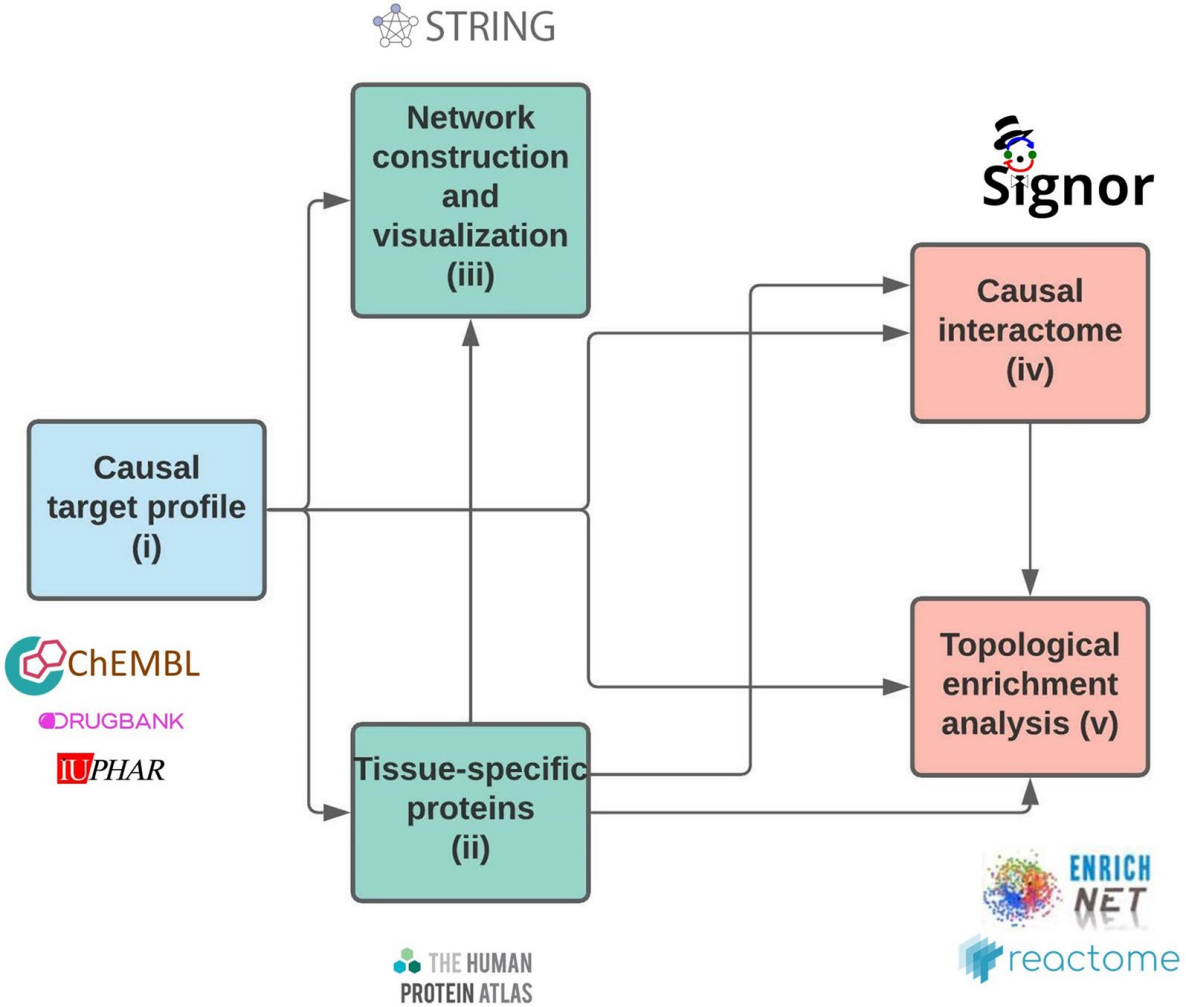
The five components of the workflow. The arrows indicate possible sequences of the building blocks; however, the combinations can be adjusted individually according to the scientific purpose.

With these 19 compounds, a first evaluation of the causal target part was performed to estimate the data availability for the drugs. After that, a single compound (CHEMBL421) was selected for further analysis. The downregulated target list of the compound was forwarded to component ii, iii iv and v, as indicated in Fig. 1. Detailed results for these two case studies are presented in the Supplement (Additional file 1).

### Workflow

The workflow has five separate components, which can be used and combined individually according to the needs of the user. In our case study, we started with (i) causal target profile and conducted (ii) for obtaining a liver-specific dataset. Parts (iii) (iv) and (v) were carried out with subsets of our data.

### Causal target data

In the first section of the workflow, causal target protein profiles can be built using three different databases. In a previous publication, a detailed description of retrieving target profiles for compounds was described [14]. However, as the previous workflow does not distinguish between positive and negative effects of the compounds, in this workflow this type of information is emphasized:

- Targets were defined as human-type single proteins, which are annotated as target proteins of the compound of interest in one of the utilized databases or
- the compound was annotated as active on the protein in biological assays or
- the compound has an activity value to the target in a pre-defined active range.

The cut-off for the active range was set to 10 μM. Our analysis was intended to be performed across diverse protein families. The activity cut-off can be adjusted to be more stringent or can be modified for particular protein families where another value might be more appropriate. Since the intention was to consider the whole systemic effect of the drug, no differentiation between therapeutic targets and putative targets were made.

#### Mode of action annotations

The ChEMBL (version 27) [15], DrugBank (version 5.1.6) [16], and IUPHAR (version 2020.4) [17] databases provide mode of action annotations on compound and target pairs. These were retrieved via programmatic access or download. Since the vocabulary of the annotations differs among the databases, an individual translation of the terms into categories was added to the workflow. The categories are summarised in Table 1.

**Table 1:** Mode of action categories defined in the workflow

Category	Description
1	Active—positive modulator
2	Active—negative modulator
3	Active—no further information
4	Inactive

Examples of the annotations and the translations of ChEMBL and IUPHAR data are shown in Tables 2 and 3, the whole annotation is provided as supplement (Additional files 2, 3).

#### CHEMBL

**Table 2:** Examples of mode of action annotations based on ChEMBL data

$action_type$ = "ACTIVATOR" = > 1
$action_type$ = "AGONIST" = > 1
$action_type$ = "ANTAGONIST" = > 2
$action_type$ = "BINDING AGENT" = > 3
$action_type$ = "BLOCKER" = > 2
$action_type$ = "MODULATOR" = > 3
$action_type$ = "NEGATIVE ALLOSTERIC MODULATOR" = > 2
$action_type$ = "NEGATIVE MODULATOR" = > 2
$action_type$ = "POSITIVE MODULATOR" = > 1
$action_type$ = "RELEASING AGENT" = > 3
$action_type$ = "STABILISER" = > 3

#### IUPHAR

**Table 3:** Mode of action annotations based on IUPHAR data

$actions$ = "Activation" = > 1
$actions$ = "Biased agonist" = > 1
$actions$ = "Binding" = > 3
$actions$ = "Competitive" = > 3
$actions$ = "Feedback inhibition" = > 2
$actions$ = "Full agonist" = > 1
$actions$ = "Inhibition" = > 2
$actions$ = "Irreversible inhibition" = > 2
$actions$ = "Mixed" = > 3
$actions$ = "Neutral" = > 3

#### Assay annotations

The ChEMBL assay API provides further possibilities to find causal targets. With this call, one can retrieve assay description data, which was used as a base for text mining. The text mining was carried out using keywords, which can imply the type of relationship between protein and compound. A list of potential keywords was trialled in KNIME, wildcards were then added to each end and the pipeline was run with the wildcard-adjusted keywords. An option to refine with exclusions could be applied to remove cases where terms from the different groups were present in the same assay but were not included at the test stage. A pChEMBL value of 5 was used as a cut-off for actives (pChEMBL >  = 5), pChEMBL 5 is equivalent to 10 μM [18]. If the annotation implied “inconclusive” or “not active”, the datapoint was removed from the list. A non-active category was added to our list for the compound-target pairs that were tested together and did not show activity. Again, the cut-off of pChEMBL 5 was used (pChEMBL < 5), and data points with the annotation of being “active” or “inconclusive” were discarded. Consequently, if the pChEMBL cut-off and the activity comment were contradictory, the assay was excluded from the analysis.

Examples of the text mining can be found in Table 4. The full annotation is available as a supplement, with the used keywords and as applied in KNIME (Additional file 4).

**Table 4:** Annotation of assay data based on assay description and keywords

Keyword	Keyword as applied in KNIME
channel blocking activity	$assay_description$ LIKE "*hannel blocking activit*" = > 2
inhibit 50%	$assay_description$ LIKE "*nhibit 50%*" = > 2
inhibiting	$assay_description$ LIKE "*nhibitin*" = > 2
inhibitor	$assay_description$ LIKE "*nhibito*" = > 2
Activation	$assay_description$ LIKE "*ctivatio*" = > 1
Channel opening activity	$assay_description$ LIKE "*hannel opening activit*" = > 1

#### Consistency

After executing the workflow with both sets of compounds, a verification step was built in to establish the consistency of the annotations of the databases and our text mining efforts. Datapoints with contradictory annotations were removed, aiming for a comprehensive and reliable analysis. For that purpose, an additional panel was added to the KNIME workflow. In this panel, the unique drug-target pairs were grouped by the annotations and every contradiction was removed from the results. For instance, if one compound-protein pair was annotated as mode of action group 1 and also 3 (Table1), the data was not removed since group 1 is a sub-group of group 3; hence there is no contradiction. However, if the compound-target pair was annotated as 1 and 2, the interaction was excluded from the analysis.

### Tissue-specificity

The workflow provides an option for tissue-specific filtering of proteins using the programmatic access of the Proteomics database ProteomicsDB (version3.0). The database aims to contribute to the identification of the human proteome, providing a large coverage [19]. Using the API call, one can retrieve the list of tissues where the protein is expressed and select the tissue of interest and create a tissue-specific sub-set. For the DILI case study, the liver was chosen as the tissue of interest.

### Identifying DILI related up- and downregulated targets

The first step in analysing the retrieved tissue-specific data for the case study was to search for proteins in both the positively and negatively modulated groups that are highly connected to the mostDILI and less or not connected to the noDILI group. For this purpose, a DILI significance score was assigned to each target protein as described below. Absolute values were used; however, the analysis can also be carried out with normalized values.

Steps of the scoring process:

1. Established to how many mostDILI and noDILI compounds the protein can be connected to, respectively.
2. If the protein can be connected to both groups, the quotient of the involvement of the two groups was calculated as follows:
  - (x)mostDILI/(x)noDILI = significance
  - (x) = number of compounds
3. The cut-off for significance was set to 5, which means the involvement of the mostDILI group is at least five times that of the noDILI group.
4. If the target was only connected to the mostDILI group, the cut-off was also set to 5, which means it is connected to at least 5 compounds.

### Network creation and visualization

The STRING database (version 11.5) aspires to collect and annotate all publicly available interactions between proteins to create a wide-ranging and unbiased global network [20]. By using the web services of STRING, the workflow was expanded with the possibility of network construction and visualization. For instance, the protein network of the proteins that are downregulated by a group of compounds can be visualized by submitting the required list of proteins. The workflow provides visualization based on the connectivity annotated in the STRING database. This allows the user to have an overview of the network and helps to identify the topological properties of the nodes. Since STRING contains different types of connections, the API call is modifiable by setting parameters such as network_flavor or network_type. In our analysis, the settings were: network_flavor = confidence, to represent the confidence score of the interaction between two nodes via the thickness of the edge, and network_type = functional, which indicates both functional and physical interactions.

### Causal protein–protein interactions

The Signaling Network Open Resource 2.0 (SIGNOR 2.0) is a public repository of causal relationship information among biological entities [21]. The database was added to the workflow as an additional layer for introducing causal protein–protein interactions. With this information, the user can have a comprehensive causal network of proteins connected to the compound(s) of interest. Here the download function provided by SIGNOR was utilized, containing information on different molecules and their connectivity. Only protein–protein type interactions were considered, and the annotated effect was extracted from the data (e.g.: upregulates.) The annotations were grouped in the same categories as discussed for the targets (Table 1).

### Topological enrichment analysis

The last part of the workflow performs TEA using the EnrichNet web application (version 1.1) [22]. EnrichNet performs graph-based statistical evaluation based on interaction networks allowing a direct interpretation of the results via their website. With this part of the workflow the possibility of different topology-based analyses is given to investigate which pathways or biological processes are enriched in the submitted data. EnrichNet offers different databases as a base for the analysis. This parameter can also be changed in the workflow. The analysis represented here was performed based on the Reactome database (version 77). Reactome is one of the major databases capturing biological pathways [23]. EnrichNet provides programmatic access, where the API call results in a link to the website of EnrichNet. This call was included in the KNIME workflow where one can carry out the enrichment analysis and open the results within the workflow via an interactive link. The results can be viewed and downloaded directly without leaving the KNIME platform.

## Results

Since different databases and a text-mining exercise were applied for creating the causal target profiles, it was considered necessary to define how consistent the annotations among the repositories were and how consistently our workflow was able to capture the information needed. The workflow initially identified 8637 unique compound-target-annotation connections, out of which 8186 were consistent and 451 have been removed. Consequently, the workflow was able to categorize 95% of the data consistently. This measure can also be seen as a reassurance of the quality of the data used since the annotations among the different databases can be considered as consistent.

### Causal target profile

The result of the causal target part of the workflow consists of three columns: ChEMBL identifier of the compound, UniProt identifier for the target proteins, and the mode of action group.

After executing the workflow with the mostDILI and noDILI compounds and applying the consistency panel, the workflow found 2987 connections for 164 (out of 180) drugs of the mostDILI group and 5199 connections for 233 (out of 272) drugs of the noDILI group.

### Tissue-specific causal targets

After applying the liver-specific protein filter, 5086 unique connections remained, 1770 for 151 mostDILI compounds and 3316 for 205 noDILI compounds. The collected, annotated, and filtered data can serve as a starting point for different types of analyses to enhance understanding of existing information. In the following section one approach is demonstrated by analysing sub-sets of the data to identify key participants and processes in connection to Drug-Induced Liver Injury.

### Identifying DILI related up- and downregulated targets

The identified tissue-specific list of proteins was analysed regarding their connectivity to the mostDILI and noDILI group to find significant proteins connected to the mostDILI compounds. The significance score was assigned as discussed in the Methods section.

Please note that the type of the analysis and the cut-offs can be selected individually depending on the corresponding analysis and considering imbalances in the dataset. The most significant proteins obtained after this exercise are summarized in Tables 5 and 6. Table 5 consists of the proteins that are significantly more often upregulated by DILI compounds. Table 6 contains the proteins that are significantly more often downregulated by the mostDILI group in comparison to the noDILI group.

**Table 5:** Most significant proteins, which are more often upregulated by the mostDILI compared to the noDILI group

Uniprot_ID	Gene_name	Significance_score
P04798	CYP1A1	5.0
P05177	CYP1A2	5.0
P08684	CYP3A4	5.0
P10275	AR	5.0

**Table 6:** Most significant proteins, which are more often downregulated by the mostDILI than the noDILI group

Uniprot_ID	Gene_name	Significance_score
P23219	PTGS1	16.0
O60656	UGT1A9	14.0
O94956	SLCO2B1	11.0
Q92887	ABCC2	9.0
P11509	CYP2A6	9.0
P22309	UGT1A1	7.5
Q9Y694	SLC22A7	7.0
P05177	CYP1A2	6.0
Q9NPD5	SLCO1B3	6.0
Q9Y6L6	SLCO1B1	6.0
P11712	CYP2C9	5.8
P02763	ORM1	5.0
P35503	UGT1A3	5.0

### Network

**Fig. 2:**
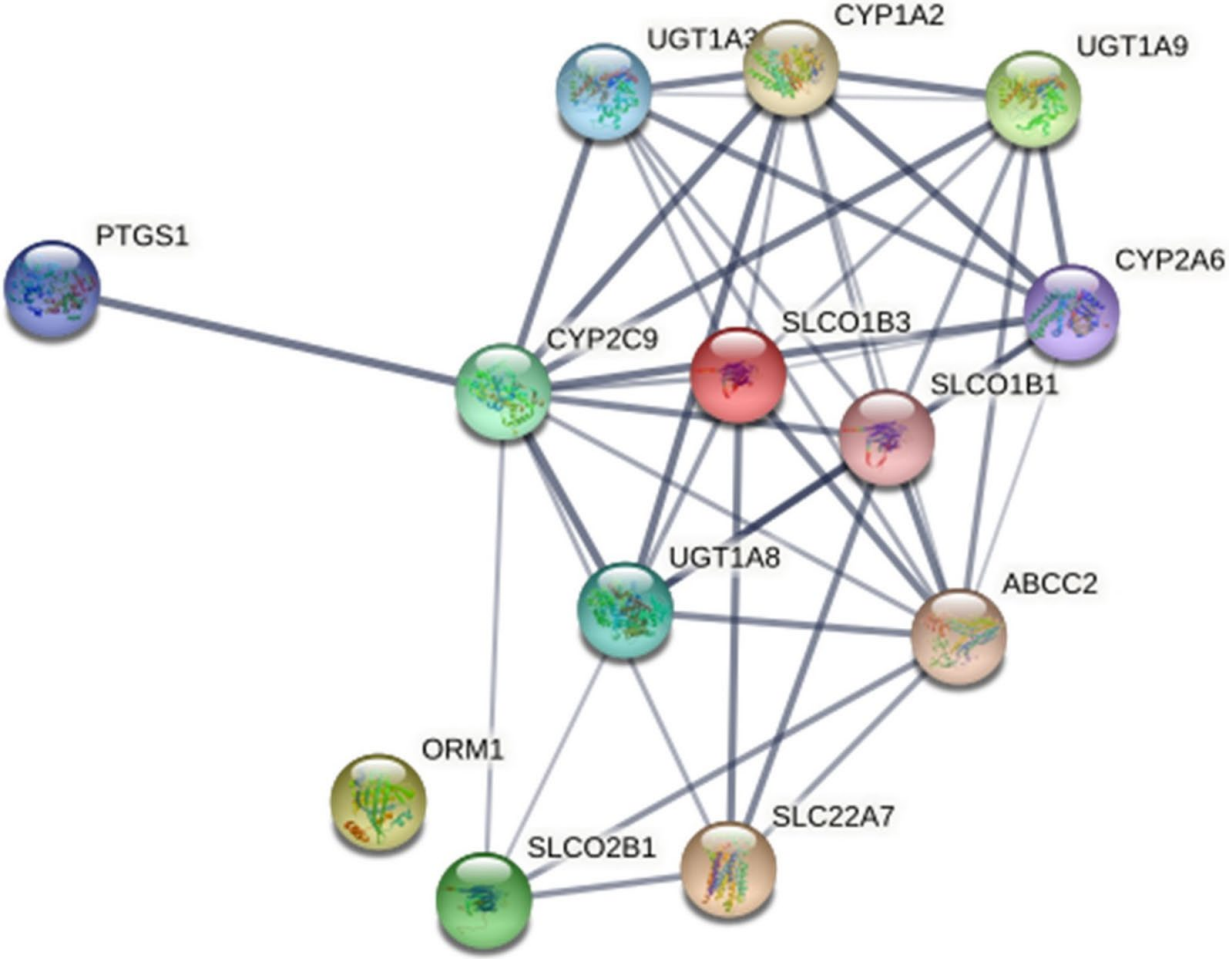
Network of significant downregulated proteins by the mostDILI group. Targets are symbolized with gene names, the thickness of the lines indicates the confidence of the interaction.

Similarly to the causal target part, the network part of the workflow also provides the possibility for different types of analyses. Here we present a subsequential analysis to the causal part, by visualizing the downregulated proteins connected significantly more often to the mostDILI group (Fig. 2).

The downregulated proteins are represented as nodes of the network, and the connections between them as edges. The thickness of the edge indicates the confidence of the protein–protein interactions.

### Causal network

The downregulated list of proteins was further analysed with the causal protein–protein interaction fragment of the workflow. The coverage of this data is sparse; however, it still can provide a base for completing the network with a causal protein–protein interactome layer. The causal connection (effect) is obtained from the SIGNOR data and the mode of interaction (moi) is assigned by the workflow, using the identical annotations as indicated in Table1. Accordingly, 1 indicatespositive modulation and 2 negative modulation. Table 7 is an example of an output row of the causal network part, with the Uniprot IDs of the target and interacting proteins, the effect, and the mode of interaction group. Table 8 summarizes the protein/interactor pairs which are significantly connected to the mostDILI group.

**Table 7:** Example of a causal network output row

target_uniprot_id	typeA	Interactor_uniprot_id	typeB	Effect	moi
O60656	protein	P20823	protein	up-regulates quantity by expression	1

**Table 8:** Upregulated proteins by the downregulated proteins significantly connected to the mostDILI group

target_uniprot_id	Interactor_uniprot_id
O60656	P20823
P22309	P35869
P22309	P20823
P22309	O75469
P22309	Q14994
P22309	P04150
Q92887	Q14653

### Enrichment analysis

With the last part of the workflow, different network-based enrichment analyses can be performed. In this section the subset of proteins, which contains the significant downregulated proteins by the mostDILI compounds (Table 6), were submitted for the TEA. In this analysis, biological pathways are also represented as graphs [24]. The results are shown in an interactive output within the KNIME workflow, where the website of EnrichNet is retrieved. The results can be viewed and downloaded directly in the workflow. The obtained results contain the pathways with a score of significance calculated by EnrichNet (XD-score) and a Fisher q-value. An XD-score higher than one is considered significant. The most significant pathways obtained with the mostDILI downregulated targets are summarized in Table 9.

**Table 9:** Result of the topological pathway analysis with the proteins summarized in Table6

Annotation (pathway/process)	XD-score	Fisher q-value
RECYCLING OF BILE ACIDS AND SALTS	1.622	0.018
GLUCURONIDATION	1.407	0.001
PHASE 1 FUNCTIONALIZATION	1.185	0.022
XENOBIOTICS	1.185	0.022

## Discussion

In the first part of the workflow (i) causal target profiles for both DILI and non-DILI groups were built. In the second (ii) part, proteins that are not expressed in the liver were filtered out. With a statistical evaluation, the most significant up- and downregulated target proteins of the mostDILI compounds were identified. Here, the most notable differences were seen at the Prostaglandin G7H synthase 1, UDP-glucuronosyltransferase 1A9, and solute carrier organic anion transporter family member 2B1. The protective effect of Prostaglandin E_2_ against harmful effects of xenobiotics in the liver is well studied [25]. However, Prostaglandin G7H synthase 1 as a key player in the unwanted event is not well documented. This finding can provide a basis for a hypothesis of how drugs can shut down the organism´s defence mechanism against toxic events. As expected, the majority of the 16 mostDILI drugs affecting Prostaglandin G7H synthase 1 are Non-steroidal anti-inflammatory drugs (NSAIDs), but not all of them (Table 10). This implies that drugs from diverse categories interfering with Prostaglandin synthesis can also have an unwanted target profile.

**Table 10:** Categories of drugs connected to Prostaglandin G7H synthase 1

Description_of_drug_indication	Compound_count
No indication provided	3
ALIMENTARY TRACT AND METABOLISM: ANTIDIARRHEALS, INTESTINAL ANTIINFLAMMATORY/ANTIINFECTIVE AGENTS: INTESTINAL ANTIINFLAMMATORY AGENTS: Aminosalicylic acid and similar agents	1
ANTIINFECTIVES FOR SYSTEMIC USE: ANTIMYCOTICS FOR SYSTEMIC USE: ANTIMYCOTICS FOR SYSTEMIC USE: Triazole derivatives	1
DERMATOLOGICALS: ANTIFUNGALS FOR DERMATOLOGICAL USE: ANTIFUNGALS FOR SYSTEMIC USE: Antifungals for systemic use	1
MUSCULO-SKELETAL SYSTEM: ANTIINFLAMMATORY AND ANTIRHEUMATIC PRODUCTS: ANTIINFLAMMATORY AND ANTIRHEUMATIC PRODUCTS, NON-STEROIDS	8
NERVOUS SYSTEM: ANALGESICS: OTHER ANALGESICS AND ANTIPYRETICS: Other analgesics and antipyretics	1
RESPIRATORY SYSTEM: DRUGS FOR OBSTRUCTIVE AIRWAY DISEASES: OTHER SYSTEMIC DRUGS FOR OBSTRUCTIVE AIRWAY DISEASES: Leukotriene receptor antagonists	1

UDP-glucuronosyltransferase 1A9 is essential for the elimination and detoxification of drugs, xenobiotics, and endogenous compounds [26]. Its inhibition can lead to perturbation in the elimination of xenobiotics resulting in accumulation and toxicity [27]. SLCO2B1 (OATP-B) is an organic anion-transporting polypeptide in the liver, which acts as an uptake transporter [28]. SLCO2B1 also mediates the Na^+^-independent transport of prostaglandins PGD2, PGE1, PGE2. Therefore, its involvement in unwanted events can be manifold.

After creating the network of the significantly downregulated group in part (iii) of the workflow, we could perceive that the subset is highly interconnected. From a network perspective, this observation can be explained by a characteristic of biological networks: highly connected nodes (so-called hubs) can also have connections in their biological roles. This hypothesis suggests that the hubs can be associated with the same disease or unwanted event [29], which would strengthen the likelihood of the proteins` potential involvement in toxicity.

The TEA of the downregulated sub-set identified four Reactome pathways that can be significantly connected to the submitted network: Recycling of bile acid and salt, Glucuronidation, Phase1 functionalisation, and Xenobiotics. Liver toxicity induced by bile-acid accumulation is well-known [30]. Glucuronidation and Phase 1 of the metabolism are involved in drug metabolism, which makes the perturbance of these pathways a plausible contributor to liver toxicity. These results indicate that the subset of network proteins can be involved in multiple processes disturbing the normal functions of metabolism and excretion of xenobiotics and potentially initiating toxic processes in the liver.

In connection to cardiotoxicity, a case study of a smaller dataset was shortly reported. Especially ion channels and serotonin receptors were significantly downregulated by the cardiotoxic group. Several of these targets are discussed in the literature in connection to cardiac diseases [31]. After filtering for the heart tissue, we discovered that the hERG potassium channel (KCH2) was also filtered out, since the Proteomics DB has no protein expression data of hERG in the heart. This highlights the main limitation of the workflow: data coverage. The workflow can only report data available in the utilized databases. Even though these databases are of high quality, their coverage also has its own limits. For instance, Proteomics DB states a coverage of 83%. Therefore, careful curation of the results cannot be avoided.

With the nephrotoxicity case study, the intention was to show the usability of the workflow with a single compound. If the data coverage is sufficient, the workflow can deliver meaningful results for one compound. Sulfasalazine (CHEMBL421) had enough causal targets to perform an analysis with the workflow and, via the enrichment analysis, significant pathways were found that can be connected to nephrotoxicity. For instance, Prostanoid hormones were already discussed in their roles in the pathogenesis of various kidney diseases [3]. For this case study, exclusion term pairs were added to the workflow, since one assay description included both terms “Inhibitors” and “Activators”. These exclusions are based on Additional file 5.

Please note that these case studies were simplified representations of the usability of the workflow, one could go more in-depth with the analysis even with the DILIRank dataset. The different components of the workflow can be individually combined or used separately.

## Conclusion and summary

Network-based approaches are valuable for understanding systemic effects of drugs. A comprehensible KNIME workflow was presented which utilizes openly available data for target and network-focused analyses. The usability of the workflow was presented by one extensive and two short case studies in connection to drug induced toxicities. The workflow was able to identify important proteins and processes that can be involved in toxic events. The pipeline is openly available and adjustable depending on the intended analysis.

## Supplementary Information

**Additional file 1:** Detailed results of the case studies of cardiac therapy and cardiotoxic drugs, as well as for CHEMBL421.

**Additional file 2:** CHEMBL mode of action annotation.

**Additional file 3:** IUPHAR mode of action annotation.

**Additional file 4:** Full assay annotation with text mining based on ChEMBL data.

**Additional file 5:** Exclusions for the text mining of assay data.

**Additional file 6:** ChEMBL identifiers of mostDILI compounds.

**Additional file 7:** ChEMBL identifiers of noDILI compounds.

**Additional file 8:** ChEMBL identifiers of cardiac therapy compounds.

**Additional file 9:** ChEMBL identifiers of cardiotoxic compounds.

**Additional file 10:** Skimmed data from DrugBank for the workflow.

**Additional file 11:** Downloaded data from the SIGNOR database.

## Data Availability

The workflow is openly available on KNIMEHub: https://hub.knime.com/barbaraf/spaces/Public/latest/~7jbMNHvalhE2ZCtU/ (Causal Network WF) with a workflow manual (causal_network_workflow_manual). The short case studies are described more in detail in Additional file 1. The assay annotation data is available in the supplementary material (Additional file 4) The analysed compounds are listed in the Additional file 6 (mostDILI compounds), Additional file 7 (noDILI compounds), Additional file 8 (cardiac_therapy compounds) and Additional file 9 (cardiotoxic compounds). Utilized web-services: https://chembl.gitbook.io/chembl-interface-documentation/web-services https://www.guidetopharmacology.org/webServices.jsp https://www.proteomicsdb.org/#api https://string-db.org/help/api/ https://signor.uniroma2.it/APIs.php https://enrichnet.org/api.html The data used for the case study is openly available from repositories referenced in the main text and were utilized via programmatic access. DrugBank datasets are released under the Creative Common’s Attribution-NonCommercial 4.0 International License and can be downloaded here: https://go.drugbank.com/releases/latest Since the mining of the whole XML file of the DrugBank data is excessive, we created a skimmed excel file with the necessary information for the workflow. This file is completely based on the downloadable part of DrugBank data, therefore copyright belongs to DrugBank https://go.drugbank.com/. The file is available as Additional file 10. The SIGNOR database provides an API for retrieving their data; however, the output is a plain text file. Therefore, we worked with the downloadable interaction tsv file, which can be downloaded here: https://signor.uniroma2.it/downloads.php and can be found as Additional file 11.

## Abbreviations

DILI: Drug-induced liver injury
FDA: U.S. Food and Drug Administration
TEA: Topological Enrichment Analysis

## References

[1] Medina-Franco JL, Giulianotti MA, Welmaker GS, Houghten RA (2013). Shifting from the single- to the multitarget paradigm in drug discovery. Drug Discov Today.

[2] Csermely P, Ágoston V, Pongor S (2005). The efficiency of multi-target drugs: the network approach might help drug design. Trends Pharmacol Sci.

[3] Reddy AS, Zhang S (2013). Polypharmacology: drug discovery for the future. Expert Rev Clin Pharmacol.

[4] Hartung T, FitzGerald RE, Jennings P (2017). Systems toxicology: real world applications and opportunities. Chem Res Toxicol.

[5] Berggård T, Linse S, James P (2007). Methods for the detection and analysis of protein–protein interactions. Proteomics.

[6] Yıldırım MA, Goh K-I, Cusick ME (2007). Drug—target network. Nat Biotechnol.

[7] Hardt C, Bauer C, Schuchhardt J, Herwig R (2018). Computational network analysis for drug toxicity prediction. Methods Mol Biol Clifton NJ.

[8] Belyaeva A, Cammarata L, Radhakrishnan A (2021). Causal network models of SARS-CoV-2 expression and aging to identify candidates for drug repurposing. Nat Commun.

[9] Ma J, Shojaie A, Michailidis G (2019). A comparative study of topology-based pathway enrichment analysis methods. BMC Bioinformatics.

[10] Agapito G, Pastrello C, Jurisica I (2021). Comprehensive pathway enrichment analysis workflows: COVID-19 case study. Brief Bioinform.

[11] Berthold MR, Cebron N, Dill F (2009). KNIME—the Konstanz information miner: version 2.0 and beyond. ACM SIGKDD Explor Newsl.

[12] Chen M, Suzuki A, Thakkar S (2016). DILIrank: the largest reference drug list ranked by the risk for developing drug-induced liver injury in humans. Drug Discov Today.

[13] Shi Y, Hua Y, Wang B (2022). In silico prediction and insights into the structural basis of drug induced nephrotoxicity. Front Pharmacol.

[14] Füzi B, Gurinova J, Hermjakob H (2021). Path4Drug: data science workflow for identification of tissue-specific biological pathways modulated by toxic drugs. Front Pharmacol.

[15] Mendez D, Gaulton A, Bento AP (2019). ChEMBL: towards direct deposition of bioassay data. Nucleic Acids Res.

[16] Wishart DS, Feunang YD, Guo AC (2018). DrugBank 5.0: a major update to the DrugBank database for 2018. Nucleic Acids Res.

[17] Harding SD, Sharman JL, Faccenda E (2018). The IUPHAR/BPS Guide to PHARMACOLOGY in 2018: updates and expansion to encompass the new guide to IMMUNOPHARMACOLOGY. Nucleic Acids Res.

[18] Bento AP, Gaulton A, Hersey A (2014). The ChEMBL bioactivity database: an update. Nucleic Acids Res.

[19] Samaras P, Schmidt T, Frejno M (2020). ProteomicsDB: a multi-omics and multi-organism resource for life science research. Nucleic Acids Res.

[20] Szklarczyk D, Gable AL, Nastou KC (2021). The STRING database in 2021: customizable protein-protein networks, and functional characterization of user-uploaded gene/measurement sets. Nucleic Acids Res.

[21] Licata L, Lo Surdo P, Iannuccelli M (2020). SIGNOR 2.0, the SIGnaling Network Open Resource 2.0: 2019 update. Nucleic Acids Res.

[22] Glaab E, Baudot A, Krasnogor N (2012). EnrichNet: network-based gene set enrichment analysis. Bioinforma Oxf Engl.

[23] Gillespie M, Jassal B, Stephan R (2021). The reactome pathway knowledgebase 2022. Nucleic Acids Res.

[24] Yang Q, Wang S, Dai E (2019). Pathway enrichment analysis approach based on topological structure and updated annotation of pathway. Brief Bioinform.

[25] Ćavar I, Kelava T, Vukojević K (2010). The role of prostaglandin E2 in acute acetaminophen hepatotoxicity in mice. Histol Histopathol.

[26] Gagné J-F, Montminy V, Belanger P (2002). Common human UGT1A polymorphisms and the altered metabolism of irinotecan active metabolite 7-ethyl-10-hydroxycamptothecin (SN-38). Mol Pharmacol.

[27] Grancharov K, Naydenova Z, Lozeva S, Golovinsky E (2001). Natural and synthetic inhibitors of UDP-glucuronosyltransferase. Pharmacol Ther.

[28] Kullak-Ublick GA, Ismair MG, Stieger B et al (2001) Organic anion-transporting polypeptide B (OATP-B) and its functional comparison with three other OATPs of human liver. Gastroenterology 120(2):525-33. 10.1053/gast.2001.21176 10.1053/gast.2001.21176 11159893

[29] Barabási A-L, Gulbahce N, Loscalzo J (2011). Network medicine: a network-based approach to human disease. Nat Rev Genet.

[30] Rodrigues AD, Lai Y, Cvijic ME (2014). Drug-induced perturbations of the bile acid pool, cholestasis, and hepatotoxicity: mechanistic considerations beyond the direct inhibition of the bile salt export pump. Drug Metab Dispos.

[31] Iqbal SM, Lemmens-Gruber R (2017). Voltage gated ion channels blockade is the underlying mechanism of BIMU8 induced cardiotoxicity. Toxicol Lett.

