# Supplementary material for: KNIME workflow for retrieving causal drug and protein interactions, building networks, and performing topological enrichment analysis demonstrated by a DILI case study
Source: J Cheminform. 2022 Jun 13;14:37. doi: 10.1186/s13321-022-00615-6 (PMC9188852; doi:10.1186/s13321-022-00615-6)
Supplement: Supplementary file 1 — Additional file 1: Detailed results of the case studies of cardiac therapy and cardiotoxic drugs, as well as for CHEMBL421. [file 13321_2022_615_MOESM1_ESM.docx]

# Methods

## Short case studies

### Cardiotoxic

To present the usability of the workflow, two additional short case studies were performed.

In the cardiotoxic case study approved, small molecules from ChEMBL with ATC Classifications Level 2 “Cardiac therapy” were downloaded, toxic compounds were filtered out. As the toxic group, withdrawn cardiotoxic compounds were collected, also from ChEMBL. The final dataset consists of 30 non-toxic cardiac therapy and 26 cardiotoxic drugs.

### Nephrotoxic

For the third case study a dataset was blended of withdrawn nephrotoxic compounds of ChEMBL and a recently published dataset of nephrotoxic compounds based-on the SIDER database [1]. The blended dataset contains 19 compounds.

With these 19 compounds a first evaluation of the causal target part was performed to estimate the data availability for the drugs. An additional panel was added for modifying contradictory assay descriptions, since one of the assay descriptions had both terms “Inhibitors” and “Activators”.

After that, one single compound (CHEMBL421) was selected to perform further components of the workflow with. The downregulated target list of the compound was forwarded to component ii, iii iv and v.

# Results

## Short case studies

### Cardiotoxicity

24 of the cardiotoxic and 28 of the cardiac therapy compounds had causal target data in the utilized databases. The most significant, frequently accruing target proteins are summarised in Table x.

| Uniprot IDs | Gene names | % of connected toxic compounds | % of connected non-toxic compounds |
| --- | --- | --- | --- |
| **Q12809** | **KCNH2** | 34.62 | 7.14 |
| **Q13936** | **CACNA1C** | 23.08 | 3.57 |
| P10635 | CYP2D6 | 19.23 | 32.14 |
| **Q14524** | **SCN5A** | 19.23 | 3.57 |
| P08684 | CYP3A4 | 15.38 | 32.14 |
| **P35498** | **SCN1A** | 15.38 | 3.57 |
| **P08183** | **ABCB1** | 15.38 | 0 |
| **P00533** | **EGFR** | 11.54 | 3.57 |
| **P06241** | **FYN** | 11.54 | 3.57 |
| P31645 | SLC6A4 | 11.54 | 7.14 |
| P35348 | ADRA1A | 11.54 | 32.14 |
| **P28223** | **HTR2A** | 11.54 | 0 |
| **P28335** | **HTR2C** | 11.54 | 0 |

Table 1: Most important downregulated target proteins by the cardiotoxic group with percentages of the involved compounds of each group. With bold proteins are highlighted, with more significant involvement by the toxic group.

The network construction via STRING with the most significant downregulated proteins in connection to the cardiotoxic group yielded an interconnected network (Figurex).


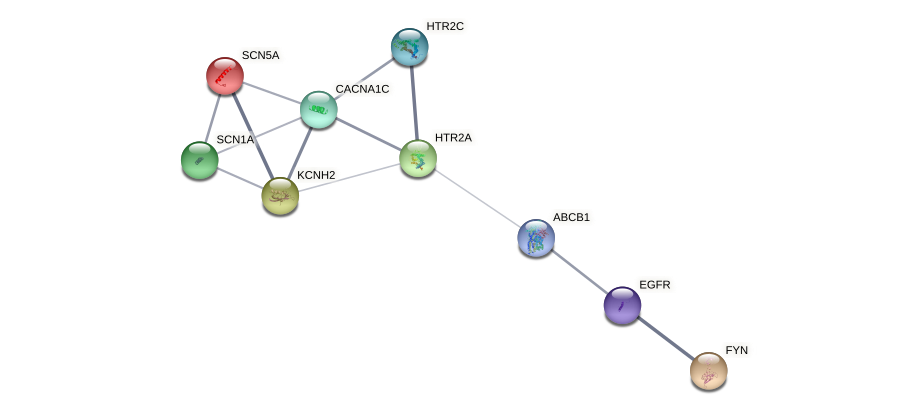


Figure 1: Network by STRING of the most significant downregulated proteins by the cardiotoxic group

The network enrichment analysis based on these 9 proteins are summarized in Table x.

| Annotation (pathway/process) | XD-score | Fisher q-value |
| --- | --- | --- |
| SEROTONIN RECEPTORS | 1.4789 | 0.0354 |
| PECAM1 INTERACTIONS | 0.8789 | 0.6524 |
| CD28 DEPENDENT VAV1 PATHWAY | 0.7971 | 0.6524 |
| GAB1 SIGNALOSOME | 0.7971 | 0.6524 |

Table x: top results of the network enrichment analysis based on the most significant downregulated proteins by the cardiotoxic group

By filtering for the heart tissue 4 of 9 proteins remained in the list: Q13936, Q14524, P00533, P06241.

However, building the causal interactome still yielded 110 interactors, since P00533, P06241 are proteins with many interactors.

### Nephrotoxicity

The causal target component returned results for 13 of 19 compounds. The distribution of the available causal targets for each compound is presented in Table x.

| CHEMBL42 | 32 |
| --- | --- |
| CHEMBL715 | 32 |
| CHEMBL1341 | 24 |
| **CHEMBL421** | **19** |
| CHEMBL16073 | 10 |
| CHEMBL956 | 5 |
| CHEMBL550348 | 4 |
| CHEMBL1460 | 3 |
| CHEMBL1467 | 3 |
| CHEMBL425 | 3 |
| CHEMBL760 | 3 |
| CHEMBL225072 | 1 |
| CHEMBL340978 | 1 |

To estimate the needed data coverage for a meaningful result, the analysis was carried out with a random compound of the middle range in regard of the target counts: Sulfasalazine (ChEMBL421).

Sulfasalazine had 19 targets, 13 of them are downregulated. Six of the downregulated targets are expressed in the kidney with 4 of them are forming a sub-network and 2 pairs are connected to each other based on the STRING database. The causal network can be extended with 220 further proteins by considering the first-degree interactors with component iv.

The network enrichment analysis based on the downregulated proteins by Sulfasalazine expressed in the kidney are summarized in Table.

| Annotation (pathway/process) | XD-score | Fisher q-value |
| --- | --- | --- |
| PROSTANOID HORMONES | 1.620 | 0.0177 |
| VIRAL DSRNA TLR3 TRIF COMPLEX ACTIVATES RIP1 | 1.4837 | 0.0177 |
| HUMAN TAK1 ACTIVATES NFKB BY PHOSPHORYLATION AND ACTIVATION OF IKKS COMPLEX | 1.1837 | 0.0188 |
| NF KB IS ACTIVATED AND SIGNALS SURVIVAL | 0.8837 | 0.4431 |
| P75NTR RECRUITS SIGNALLING COMPLEXES | 0.8837 | 0.4431 |

Table 2: top results of the network enrichment analysis based on downregulated proteins expressed in the kidney by Sulfasalazine

# Discussion

In connection to cardiotoxicity, a case study of a smaller dataset was shortly reported. Especially ion channels and serotonin receptors were significantly downregulated by the cardiotoxic group. Several of these targets are discussed in the literature in connection to cardiac diseases [2], [3]. After filtering for the heart tissue, the observation was made, that the hERG potassium channel (KCH2) was also filtered out, since the Proteomics DB has no protein expression data of hERG in the heart. This fact points us to the main limitation of the workflow: data coverage. The workflow can only report data available in the utilized databases. Even though they are of high quality, their coverage has also its own limits. For instance, Proteomics DB states a coverage of 83 %. Therefore, careful curation of the results cannot be avoided.

With the nephrotoxic case study, the intention was to show the usability of the workflow with one single compound. If the data coverage is efficient, the workflow can deliver meaningful results for one compound. Sulfasalazine (CHEMBL421) had enough causal targets to perform an analysis with the workflow and via the enrichment analysis significant pathways were found that can be connected to nephrotoxicity. For instance, Postanoid hormones were already discussed in their roles in the pathogenesis of various kidney diseases [4].

# References

1. Shi Y, Hua Y, Wang B, et al (2022) In Silico Prediction and Insights Into the Structural Basis of Drug Induced Nephrotoxicity. Front Pharmacol 12:

2. Iqbal SM, Lemmens-Gruber R (2017) Voltage gated ion channels blockade is the underlying mechanism of BIMU8 induced cardiotoxicity. Toxicol Lett 277:64–68. https://doi.org/10.1016/j.toxlet.2017.05.024

3. Zhang Q, Chen J, Qin Y, et al (2018) Mutations in voltage-gated L-type calcium channel: implications in cardiac arrhythmia. Channels 12:201–218. https://doi.org/10.1080/19336950.2018.1499368

4. Li Y, Xia W, Zhao F, et al (2018) Prostaglandins in the pathogenesis of kidney diseases. Oncotarget 9:26586–26602. https://doi.org/10.18632/oncotarget.25005
